# Are Isomeric Alkenes Used in Species Recognition among Neo-Tropical Stingless Bees (*Melipona* Spp)

**DOI:** 10.1007/s10886-017-0901-5

**Published:** 2017-11-17

**Authors:** Stephen J. Martin, Sue Shemilt, Cândida B. da S. Lima, Carlos A. L. de Carvalho

**Affiliations:** 1 School of Environment and Life Sciences, The University of Salford, M5 4WT, Manchester, UK; 2 Chemical Ecology Group, School of Physical and Geographical Sciences, Lennard-Jones Laboratory, Keele University, Newcastle upon Tyne, ST5 5BG UK; 3 Programa de Pós Graduação em Ciências Agrárias, Universidade Federal do Recôncavo da Bahia, Rua Ruí Barbosa, 710 - Centro, Cruz das Almas, BA 44380-000 Brazil

**Keywords:** Stingless bees, *Melipona*, Cuticular hydrocarbons, Alkenes, Dimethyldisulfide, Chemical communciation

## Abstract

Our understanding of the role of cuticular hydrocarbons (CHC) in recognition is based largely on temperate ant species and honey bees. The stingless bees remain relatively poorly studied, despite being the largest group of eusocial bees, comprising more than 400 species in some 60 genera. The Meliponini and Apini diverged between 80–130 Myr B.P. so the evolutionary trajectories that shaped the chemical communication systems in ants, honeybees and stingless bees may be very different. The aim of this study was to study if a unique species CHC signal existed in Neotropical stingless bees, as has been shown for many temperate species, and what compounds are involved. This was achieved by collecting CHC data from 24 colonies belonging to six species of *Melipona* from North-Eastern Brazil and comparing the results with previously published CHC studies on *Melipona*. We found that each of the eleven *Melipona* species studied so far each produced a unique species CHC signal based around their alkene isomer production. A remarkable number of alkene isomers, up to 25 in *M. asilvai,* indicated the diversification of alkene positional isomers among the stingless bees. The only other group to have really diversified in alkene isomer production are the primitively eusocial Bumblebees (*Bombus* spp), which are the sister group of the stingless bees. Furthermore, among the eleven Neotropical *Melipona* species we could detect no effect of the environment on the proportion of alkane production as has been suggested for some other species.

**Electronic supplementary material:**

The online version of this article (10.1007/s10886-017-0901-5) contains supplementary material, which is available to authorized users.

## Introduction

Chemical communication is the oldest form of communication across all forms of life (Wilson 1970). Pheromones are one of the most important chemical signals (Wyatt 2013), and are particularly well-studied in insects (Howard and Blomquist 2005). Short-range contact semiochemicals are used by many insects to identify other individuals of the same or different species (Wyatt 2013) and cuticular hydrocarbons (CHC) are an important group of compounds used in recognition (Blomquist and Bagnères 2010; Wyatt 2013). This lipid layer preserves the insect from desiccation, cuticle abrasion and infection, thus directly ensuring their survival (Lockey 1988). However, across many insect taxa, cuticular lipids have evolved to become part of their communication system by enabling them to differentiate between friend and foe or find a mate (Blomquist and Bagnères 2010; Prestwich and Blomquist 2014).

Currently much of our understanding of CHC’s is based on temperate ant species and honeybees (Breed and Bennett 1987; Kather and Martin 2015; Pradella et al. 2015; Tannure-Nascimento et al. 2008, 2009). The stingless bees (*Meliponini*) and the honey bees (*Apini*) are the only two groups of highly eusocial bees. The well-studied honey bees have only 11 species all within a single genus (*Apis*), whereas the stingless bees remain relatively poorly studied (Leonhardt 2017), despite being the largest group of eusocial bees, comprising more than 400 species in some 60 genera (Rasmussen and Cameron 2010) and are responsible for pollinating 40–90% of native flora in some regions of Brazil (Nascimento et al. 2000). The *Apini* and *Meliponini* diverged between 80 and 130 Myr B.P. (Michener and Grimaldi 1988; Rasmussen and Cameron 2010) and so ants, honeybees and stingless bees have very different evolutionary trajectories that will have shaped their chemical communication systems.

The majority of stingless bees occur in the Neo-tropics (Roubik 1989) with colonies typically containing 200–700 adults (Sakagami 1982) and they have a perennial life-cycle (Michener 2000). During their evolution, stingless bees have become adapted to inhabit a wide range of climatic environments. For example, in Brazil, *Melipona fasciculata* and *M. mandacaia* inhabit very hot and arid areas in Caatinga and Cerrado, whereas *M. scutellaris* and *M. quadrifasciata* occur in humid Atlantic forests. Therefore, this group are ideally suited to look for the effect of the environment on their CHC profiles.

The aim of this investigation was to collect new data on the CHC profiles in six species of *Melipona* from across North-eastern Brazil. This new data was compared with that from all published CHC studies on *Melipona* to investigate if a stable species CHC signal exists in this group. Finally, we used the entire dataset to determine which CHC groups might be used in species recognition and what role the environment is playing in shaping the CHC profiles in stingless bees in South America.

## Methods and Materials

### Stingless Bee Samples

**Fig. 1:**
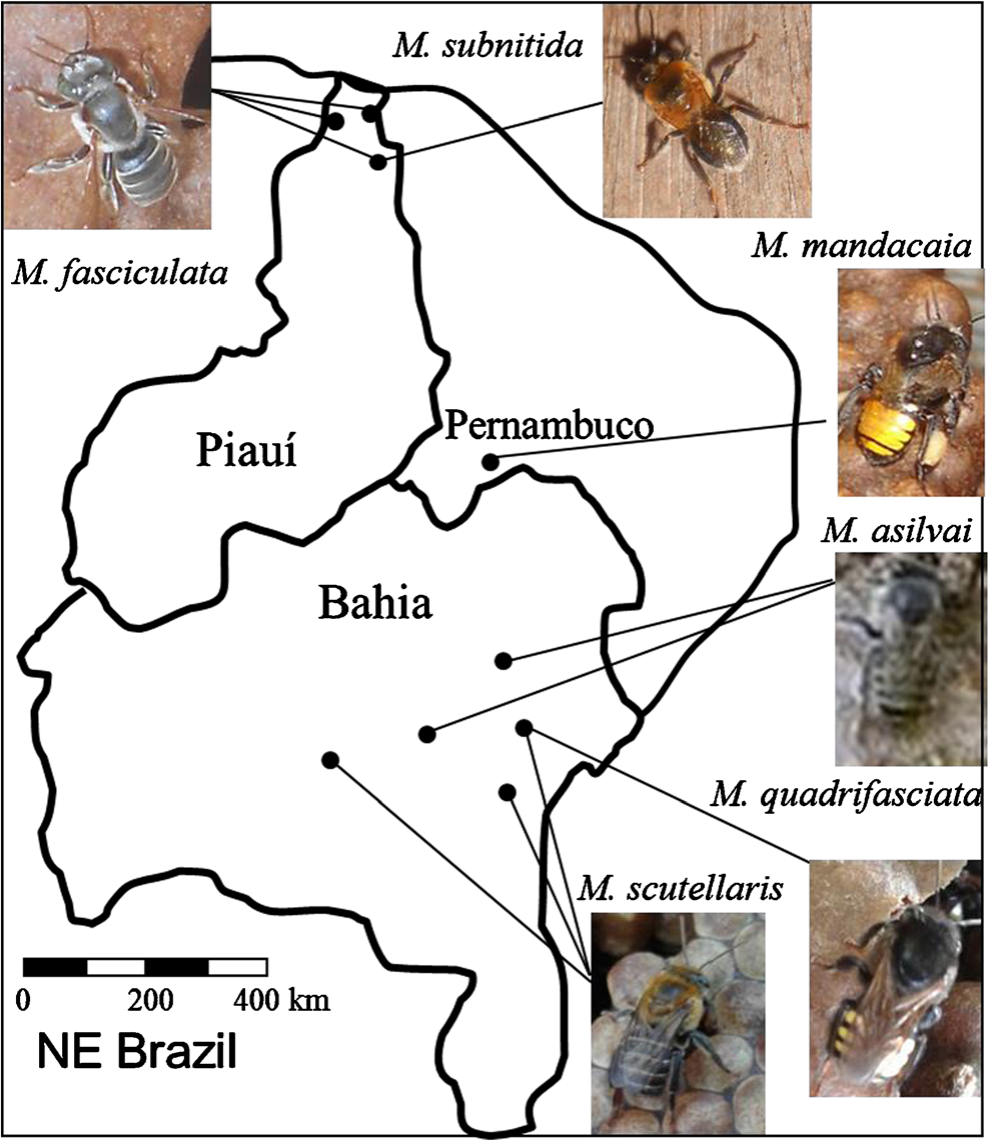
Geographic location and images of the six species of *Melipona* collected across the states of Piauí, Pernambuco and Bahia in North-eastern Brazil

All bees were collected from meliponaries maintained by local people across NE Brazil in the states of Bahia, Pernambuco and Piauí (Fig. 1). Adult workers were collected by placing a clear tube over the colony entrance and tapping the nest box lightly. All colonies were sampled between October 2014 and March 2017.

In order to determine the amount of intra- and inter-colony variation in CHC profiles we initially studied *M. fasciculata* by sampling 10 workers from each of the six colonies in October 2014. The colonies were located in three different meliponaries that were between 5 and 70 km apart (Fig. 1). All three locations were in the hot and arid area with average temperatures of 25 °C – 28 °C and low rainfall of around 20 to 40 cm a year. This established that very low levels of intra or inter-colony variation existed across the 53 individuals studied (Supplemental Fig. S1). Therefore, we subsequently collected five individuals from three to six colonies of *M. scutellaris, M. asilvai*, *M. quadrifasciata* and *M. mandacaia,* depending on their availability. From a single rare *M. subnitida* colony we collected ten individuals*.*

### Chemical Analysis

As in previous CHC studies of stingless bees (e.g. Abdalla et al. 2003; Kerr et al. 2004) we removed both sets of wings from each bee and placed them in a glass vial with 50 μl of HPLC grade hexane. After 10 min, the wings were removed and the hexane evaporated to dryness. Vials were then sealed and stored at 5 °C until just prior to analysis when HPLC grade hexane (30 μl) was added to each vial.

Samples were analyzed on an Agilent 7890 gas chromatograph (GC) connected to an Agilent 5975 MSD quadruple mass spectrometer (Agilent, Stockport, Cheshire, UK) operated in electron impact mode at 70 eV. The GC was equipped with a Vf-5ht UltiMetal column (30 m × 0.25 mm i.d. × 0.1 μm film thickness; Agilent) and the oven temperature was programmed from 70 to 200 °C at 40 °C min^−1^, then from 200 to 320 °C at 25 °C min^−1^ and held for 2 min at 320 °C. Carrier gas was helium at a constant flow rate of 1.0 ml min^−1^. For each sample 2 μl were injected in the splitless mode.

The data were analyzed using Agilent ChemStation and standard MS databases, diagnostic ions and their Kovats Indices for compound identification. The positions of alkene double bond were determined using dimethyl disulfide (DMDS) derivatization (Carlson et al. 1989). For this the wing extracts were pooled by colony (*M. asilvai*, *M. quadrifasciata, M. mandacaia, M. scutellaris* and *M. subnitida)* or location (*M. fasciculata*) then subjected to DMDS derivatization and re-analyzed on the GC-MS under similar conditions to those above. This resulted in 21 derivatized samples.

The configuration of the double bonds in the alkenes was not determined, but was assumed to be (*Z*) due to their presumed biosynthesis via the action of desaturase enzymes on saturated fatty acids (Morgan 2010).

### Published *Melipona* CHC Studies

**Fig. 2:**
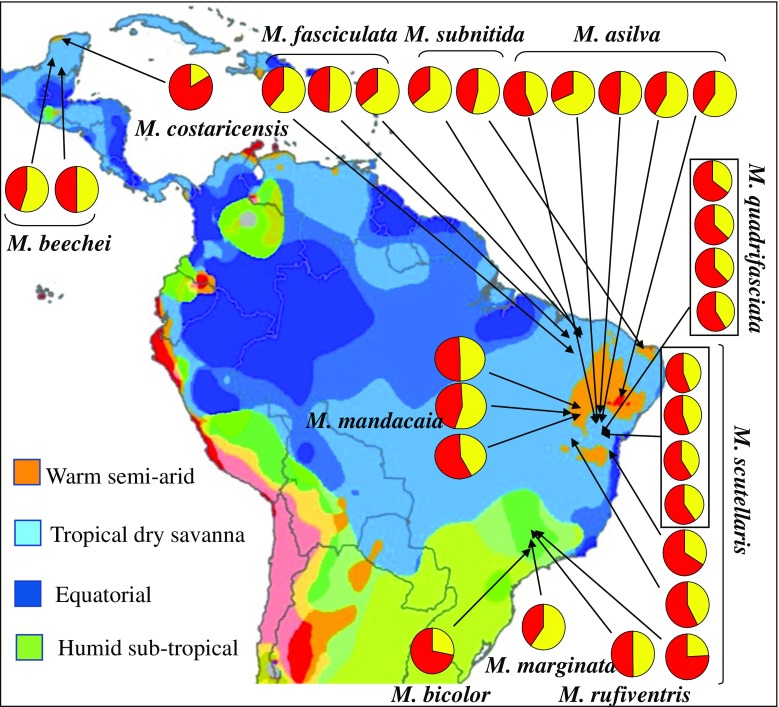
Proportion of alkanes (yellow) and olefins (red) for eleven *Melipona* species of stingless bees that have been sampled in four different climatic zones across South America. The prediction that the proportion of alkanes would increase from humid to semi-arid areas is not supported. See Table 1 for source data

A review of the literature indicated only eight previous studies (Abdalla et al. 2003; Ferreira-Caliman et al. 2010; Kerr et al. 2004; Leonhardt et al. 2013; Nascimento and Nascimento 2012; Pianaro et al. 2007;dos Santos et al. 2015; Quezada-Euan et al. 2013;) that have investigated a total of ten species of *Melipona*, all which occur in South America (Fig. 2). The data from Kerr et al. (2004) was of limited value since the GC-MS column was only heated to 250 °C rather than the normal 320 °C, resulting in the CHC above nonacosane (C_29_) probably not being detected. Although Leonhardt et al. (2013) only reported the proportions and number of compound groups these authors kindly supplied the raw data so *M. costaricensis* and *M. beecheii* could be included in the analysis.

### Statistical Analysis

A total of 134 good quality profiles were obtained, for which the peak area (Total Ion Count, TIC) of each compound was converted into a percentage of the total TIC for each bee. The average and standard deviation was then calculated for each compound for each species. To estimate the proportions of the various alkene isomers which elute closely together, the presence or absence of each isomer was determined in analyses of the DMDS derivatives (Supplemental Fig. S2–S6), then using their relative strengths we were able to identify most of alkene isomers and integrate them in the analyses of the underivatized alkenes (Supplemental Fig. S7). Where it was not possible to separate isomers this is indicated.

## Results

### Species CHC Profiles

In all six species their profiles were dominated by a series of *n*-alkanes and olefins (alkenes and dienes) ranging in chain lengths from C_23_ to C_33_ depending on the species (Tables 1 and 2). Although all species produced basically the same alkanes, the number, chain length and positional isomers of the alkenes were unique to each species and shared by all individuals within that species, irrespective of location or time of collection. Across the six-species studied, ten alkene isomers (Tables 1 and 2) were detected with each species having a different number of alkene isomers (Table 2). Some species like *M. fasciculata* had only one positional isomer, i.e. (*Z*)-9, whereas *M. asilvai* had nine isomers. Furthermore, each alkene isomer including the more prevalent isomers, such as (Z)-9, has a species-specific distribution across the various change lengths (Table 1). Therefore, all six species of *Melipona* studied had their own very distinctive CHC profile even if this was just based on their alkene profile.

**Table 1:** Means and standard deviations of the percentages of each cuticular hydrocarbon in six species of *Melipona* stingless bees investigated in this study

Compound	*M. fasciculata* (*N* = 53)	*M. quadrifasciata* (*N* = 18)	*M. scutellaris* (*N* = 23)	*M. asilvai* (*N* = 16)	*M. subnitida*(*N* = 10)	*M. mandacaia* (*N* = 14)
(*Z*)-9-Tricosene			0.2 ± 0.1			
Tricosane		**2.1 ± 0.8**	**2.9 ± 0.8**	**1.1 ± 0.3**	**0.8 ± 0.4**	**2.4 ± 0.4**
(*Z*)-11-Pentacosene			0.1 ± 0.1	0.1 ± 0.1		
(*Z*)-10-Pentacosene					1.5 ± 0.4	
(*Z*)-9-Pentacosene		2.1 ± 0.8	6.7 ± 2.5	0.5 ± 0.3	2.4 ± 0.6	33.6 ± 6.2
(*Z*)-7-Pentacosene			0.5 ± 0.5	1.1 ± 0.7		1.3 ± 0.4
(*Z*)-6-Pentacosene					1.9 ± 0.5	
Pentacosane	**14 ± 3.3**	**14.9 ± 1.7**	**21.2 ± 3.3**	**29 ± 5.8**	**36 ± 4.1**	**29.9 ± 5.9**
9,11-Methylpentacosane			0.6 ± 0.3			
5-Methylpentacosane		0.5 ± 0.1				1 ± 0.2
3-Methylpentacosane		1.3 ± 0.1				1 ± 0.1
Heptacosadiene				0.1 ± 0.0		
(*Z*)-13-Heptacosene				0.2 ± 0.3		
(*Z*)-11-Hexacosene			0.1 ± 0.1			
(*Z*)-10-Heptacosene				0.4 ± 0.4	0.8 ± 0.3	
(*Z*)-9-Heptacosene	6.2 ± 2.6	45 ± 2.2	22 ± 3.4	0.4 ± 0.3	1.9 ± 0.6	7.1 ± 1.1
(*Z*)-8-Heptacosene				0.7 ± 0.3		
(*Z*)-7-Heptacosene			1 ± 0.6	2.2 ± 1.5		0.4 ± 0.1
(*Z*)-6-Heptacosene					0.4 ± 0.3	
Heptacosane	**32.9 ± 9.7**	**14.5 ± 1.4**	**11.9 ± 3**	**6.9 ± 1.4**	**22.3 ± 3.0**	**7.8 ± 1.7**
9,11-Methylheptacosane		0.1 ± 0.1	0.7 ± 0.1			
7-Methylheptacosane		0.3 ± 0.1				
5-Methylheptacosane		0.4 ± 0.1				0.8 ± 0.2
3-Methylheptacosane						
Nonacosadiene			0.1 ± 0.1	0.5 ± 0.3	2.3 ± 0.5	
Nonacosadiene				0.9 ± 0.5	0.7 ± 0.3	
(*Z*)-13-Nonacosene			0.1 ± 0.1			
(*Z*)-12 + 13-Nonacosene				1.2 ± 0.7		
(*Z*)-10-Nonacosene				7.9 ± 1.1	2.4 ± 0.6	
(*Z*)-9-Nonacosene	30.2 ± 11	12.1 ± 1.1	6.8 ± 2.2		14.6 ± 3.5	4.9 ± 0.8
(*Z*)-8-Nonacosene				1.6 ± 0.4	0.3 ± 0.2	
(*Z*)-7-Nonacosene			0.6 ± 0.3	6.0 ± 3.3	0.7 ± 0.3	0.5 ± 0.1
(*Z*)-6-Nonacosene					2.3 ± 0.5	
Nonacosane	**9.6 ± 2.9**	**4 ± 0.6**	**3.6 ± 1.1**	**12.8 ± 5.5**	**4.9 ± 0.8**	**6.1 ± 1.0**
9,11-Methylnonocosane			0.7 ± 0.2	1.2 ± 0.1		
7-Methylnonocosane					5.7 ± 1.3	
Hentriacontadiene				1.1 ± 0.7	1.2 ± 0.4	
Hentriacontadiene			0.2 ± 0.1	2.2 ± 1.6	1.6 ± 0.4	
(*Z*)-15-Hentriacontene				0.1 ± 0.1		
(*Z*)-13-Hentriacontene			0.3 ± 0.1			
(*Z*)-12 > 14> > 15-Hentriacontene				8.8 ± 2.7		
(*Z*)-11-Hentriacontene				0.1 ± 0.1		
(*Z*)-10-Hentriacontene				3.8 ± 1.1	1.6 ± 0.4	
(*Z*)-9-Hentriacontene	4.5 ± 1.6	1.9 ± 0.4	15.3 ± 3.5	0.1 ± 0.1	1.0 ± 0.2	1.2 ± 0.3
(*Z*)-7-Hentriacontene				2.8 ± 1.3	3.2 ± 1.0	
Hentriacontane	**2.2 ± 0.6**	**1.1 ± 0.4**	**0.9 ± 0.2**	**5.7 ± 3.4**	**1.2 ± 0.4**	**2.1 ± 0.4**
9,11-Methylhentriacontane			1.0 ± 0.3			
Tritriacontadiene			0.5 ± 0.1	0.3 ± 0.3		
Tritriacontadiene			0.9 ± 0.3			
(*Z*)-16 > 14 > 12 > 10-Tritriacontene				0.9 ± 0.9		
(*Z*)-9-Tritriacontene		0.1 ± 0.3	1.1 ± 0.4			

The numbers of individual workers from which high quality total ion chromatograms were obtained are given in parenthesis. The compounds are given in retention order and those in bold are the *n*-alkanes. Were it is not possible to separate out closely eluting isomers the relative abundances of the isomers are given in order of abundance based on the DMDS ion counts

**Table 2:** Comparison of the number of alkene and (diene) isomers at each chain length for each of the six *Melipona* species studied here and those in other published studies

Species	C_23:1_	C_25:1_ (C_25:2)_	C_27:1_ (C_27:2)_	C_29:1_ (C_29:2)_	C_31:1_ (C_31:2)_	C_33:1_ (C_33:2)_	Total
*M. asilvai (*This study*)* ([Z]-16,15,14,12,11,10,9,8,7)	1	3	5(1)	5(2)	7(2)	4(1)	25(6)
*M. aslivai* (Nascimento and Nascimento 2012)	2	3	3(2)	4(2)			15(9)
*M.beecheii* (Quezada-Euan et al. 2013)		1	1	1	2(1)	1(1)	6(2)
*M.beecheii* (Leonhardt et al. 2013)	1	4		3(1)	2		9(1)
*M. bicolor* (Abdalla et al. 2003)		1	1	1			3
*M. costarisensis* (Leonhardt et al. 2013)	3	4(1)		4(2)	3(4)	1(1)	15(8)
*M. fasciculata* (This study) **(** ***Z*** **)-9**			1	1	1		3
*M. mandacaia* (This study) **(** ***Z*** **)-9,7**		2	2	2	1		7
*M. marginata (*Ferreira-Caliman et al. 2010 *)*	1	3	3	2(2)	4(2)		13(4)
*M. quadrifasciata* (This study) **(** ***Z*** **)-9,14**		1	1	2	1	1	6
*M. rufiventris* (Pianaro et al. 2007)	2	2	2	2(1)	2(3)	2(2)	12(6)
*M. scutellaris* (Pianaro et al. 2007)		2	2	2(1)	1	1	8(1)
*M. scutellaris* (This study) **(** ***Z*** **)-9, 11,13**	1	2	3	3	2(1)	1(2)	12(3)
*M. subnitida* (dos Santos et al. 2015)	1	3	3	3(2)	2(2)		12(4)
*M. subnitida* (This study) **(** ***Z*** **)-10,9,8,7,6**		3	2	3(2)	2(2)		10(4)
Totals	12	34(1)	30(3)	37(15)	30(17)	11(7)	

The alkene isomers determined for the first time in this study are given in bold

These species-specific CHC profiles remained when we increased the data set to include all previously published data (Table 2). Despite maximum distances between study sites (*M. asilvai* 407 km; *M. subnitida* 751 km; *M. scutellaris* 1172 km) the same or similar species-specific patterns of olefins were consistently found in all populations in these three species.

We also investigated if we could find any colony-specific differences in their CHC profiles in any of the six species. Clear, quantitative colony-specific differences in some of the alkene-isomers (Z-7-C_27_, Z-10-C_29_, Z-12 + 14-C_31_) could be seen between colonies of *M. asilvai*, but not in any of the other species.

### Effect of the Environment on CHC Profile

We used all available data to look for any association between the proportion of alkane production in a species and the climatic conditions where they were collected (Fig. 2). This indicated that no clear pattern emerged despite samples being analyzed for several species that live in several very different climatic zones (Fig. 2).

## Discussion

Each CHC profile of the eleven *Melipona* species studied so is unique, even if based only on their alkene isomer production (Table 2). A remarkable number of alkene isomers were obtained from just the six species in which we determined the positional isomers. For example, *M. asilvai* had nine positional isomers (*Z*)-16, (*Z*)-15, (*Z*)-14, (*Z*)-12, (*Z*)-11, (*Z*)-10, (*Z*)-9, (*Z*)-8, and (*Z*)-7, which when combined with different carbon chain lengths resulted in 25 alkene isomers being produced (Table 1). This is the highest number isomers found in any species of Hymenoptera so far studied (Kather and Martin 2015). The only other group of insects to have really diversified in production of alkene isomers is the primitively eusocial Bumblebee (*Bombus* spp). In this group up to nine positional alkene isomers from (*Z*)-5 to (*Z*)-21 are produced, again in unique, species-specific, stable patterns (Martin et al. 2010). In evolutionary terms, bumblebees (*Bombini*) are the sister group of the stingless bees (*Meliponini*) (Koulianos et al. 1999) and this may help explain why these groups have diversified their alkene production relative to all other groups of Hymenoptera (Kather and Martin 2015).

Despite the diverse isomer patterns detected in this study, the reported number of alkenes in three species that had been previously studied were very similar to each of those species is this study (Table 2). This was despite being collected in totally different locations or climate zones (Fig. 2). This stability of the species specific CHC profile over large geographical distances has also been found in *Formica* (Martin et al. 2008) and *Myrmica* ants (Guillem et al. 2016). This helps explain why environmental factors appeared to have little or no impact on qualitative CHC profile. Leonhardt et al. (2013) also demonstrated that alkanes, alkenes and alkadienes showed no or little correlation with the geographical distribution of stingless bees from Southeast Asia, Australia and Central America. It has been suggested in *Drosophila* flies that the climate affects the proportions of alkanes produced (Rouault et al. 2001), but we were unable to detect any such effect among the Neotropical stingless bees (Fig. 2). Furthermore, in *M. bicolor* the proportion of alkanes in the CHC profile varied from 74% in nurse workers to just 28% in foraging workers (Abdalla et al. 2003), whereas, in *M. marginata* the opposite situation was reported (alkanes = 45% and 60% in nurses and foragers respectively) (Ferreira-Caliman et al. 2010). Thus results can be influenced by the age of bee sampled and task it is performing since this affects the proportion of alkanes produced (Kather et al. 2011; Martin and Drijfhout 2009).

The importance of alkenes as putative recognition cues is supported by the production of large proportions of alkenes, coupled with their diversification. Although beyond the scope of this study, colony-specific differences were seen in the some of the alkene-isomers in *M. asilvai* and may help explain the nest-mate recognition that has been shown in this species (Nascimento and Nascimento 2012) and merits further study. A central problem in chemical ecology is that the sensitivity of insects to chemical change can be much greater than we are able to detect with even our best methods (Angioy et al. 2003). Unless the variation in colony specific signals is large, as in *Formica exsecta*, for example (Martin et al. 2013) they are difficult to detect as has been the case in the honey bee (*Apis mellifera*) (Pradella et al. 2015).

In the Old-World tropics (Borneo) six species of stingless bees were studied by Leonhardt et al. (2009, 2011) and each species had a distinct chemical profile based on both genetically determined compounds (CHC’s) and environmentally derived terpenes, collected from tree resins that help protect social insect colonies from micro-organism and pathogens (Brütsch et al. 2017). Several of these Old-World species also produced a high proportion of alkenes although their isomeric composition has yet to be determined (Leonhardt 2017). Based on this study, we would predict a wide range of alkene-isomers to be found among stingless bees both in the Old and New World, since it appears that in general bees have specialized in alkene diversity, rather than in the diversity of methyl-branching found in the ants and wasps (Kather and Martin 2015).

## Electronic supplementary material

ESM 1 (PDF 33 kb)

ESM 2 (PDF 104 kb)

ESM 3 (PDF 134 kb)

ESM 4 (PDF 93.2 kb)

ESM 5 (PDF 91.5 kb)

ESM 6 (PDF 127 kb)

ESM 7 (PDF 73 kb)
