# Supplementary material for: Are Isomeric Alkenes Used in Species Recognition among Neo-Tropical Stingless Bees (Melipona Spp)
Source: J Chem Ecol. 2017 Nov 17;43(11):1066–72. doi: 10.1007/s10886-017-0901-5 (PMC5735199; doi:10.1007/s10886-017-0901-5)
Supplement: Supplementary file 1 — (PDF 33 kb) [file 10886_2017_901_MOESM1_ESM.pdf]

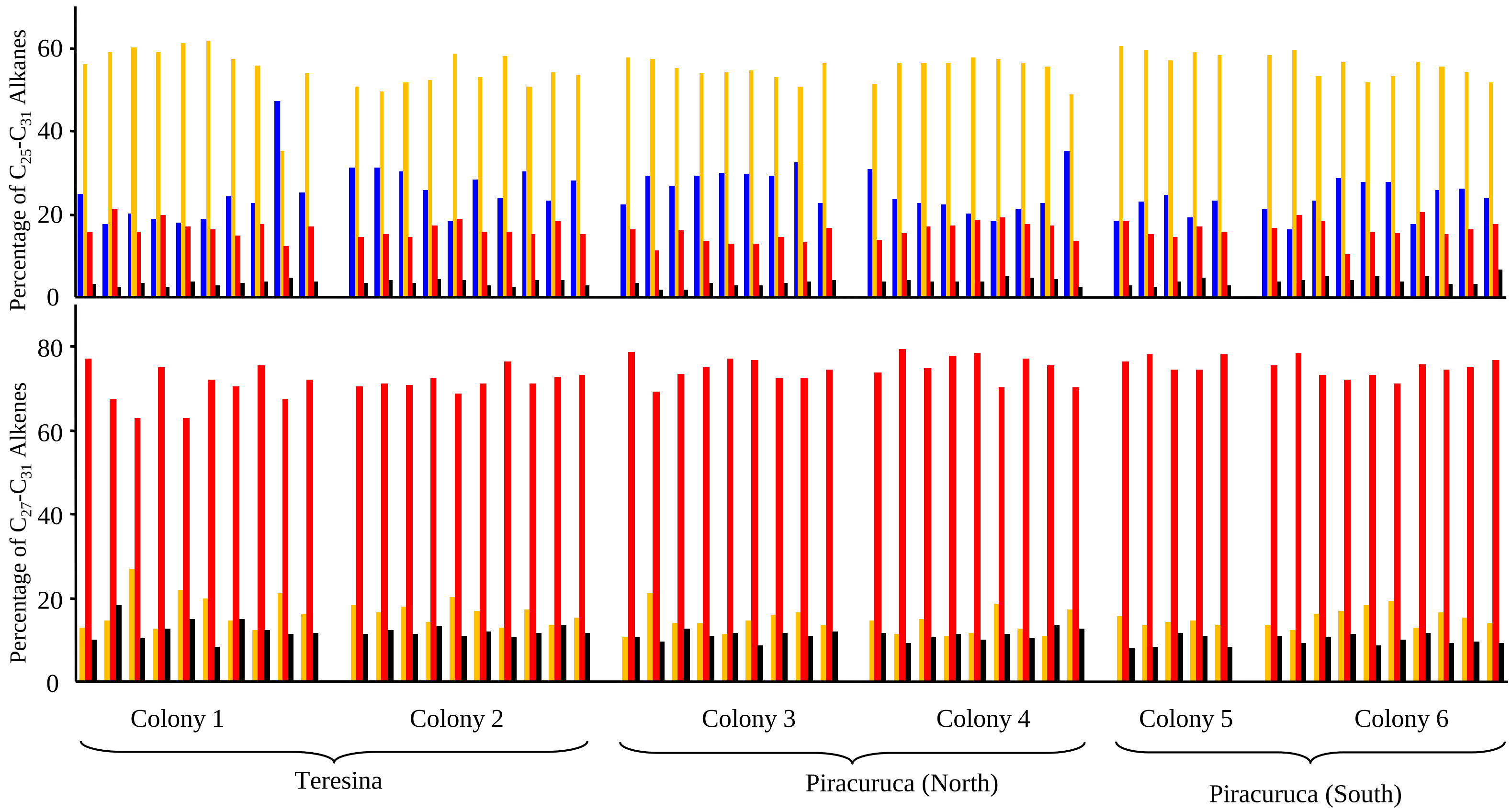

S1. Proportion of alkanes (upper) and alkenes (lower) of 53 individual *Melipona fasciculata* from six colonies located in 3 meliponaires. Each carbon chain length has a different colour with C25 =blue, C27 = yellow, C29 = red and C31 = black.
