# Supplementary material for: Are Isomeric Alkenes Used in Species Recognition among Neo-Tropical Stingless Bees (Melipona Spp)
Source: J Chem Ecol. 2017 Nov 17;43(11):1066–72. doi: 10.1007/s10886-017-0901-5 (PMC5735199; doi:10.1007/s10886-017-0901-5)
Supplement: Supplementary file 2 — (PDF 104 kb) [file 10886_2017_901_MOESM2_ESM.pdf]

M. fasciculata

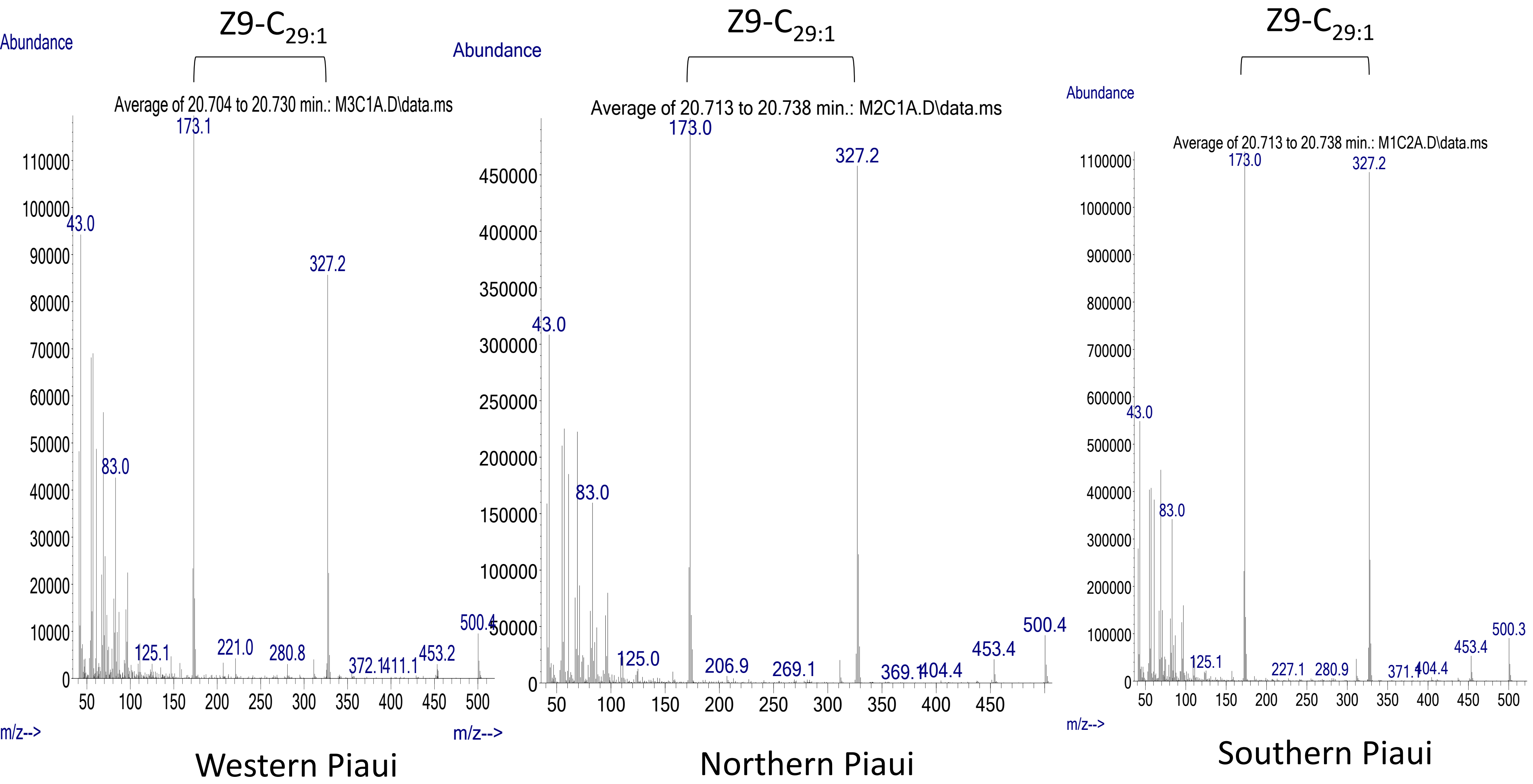

Fig. S2. The fragmentation patterns after a DMDS reaction showing the paired ions associated with the Z9 alkene isomer in *M. fasciculate* from three different locations across the Brazilian State of Piaui.
