# Supplementary material for: Are Isomeric Alkenes Used in Species Recognition among Neo-Tropical Stingless Bees (Melipona Spp)
Source: J Chem Ecol. 2017 Nov 17;43(11):1066–72. doi: 10.1007/s10886-017-0901-5 (PMC5735199; doi:10.1007/s10886-017-0901-5)
Supplement: Supplementary file 4 — (PDF 93.2 kb) [file 10886_2017_901_MOESM4_ESM.pdf]

M. quadrifasciata

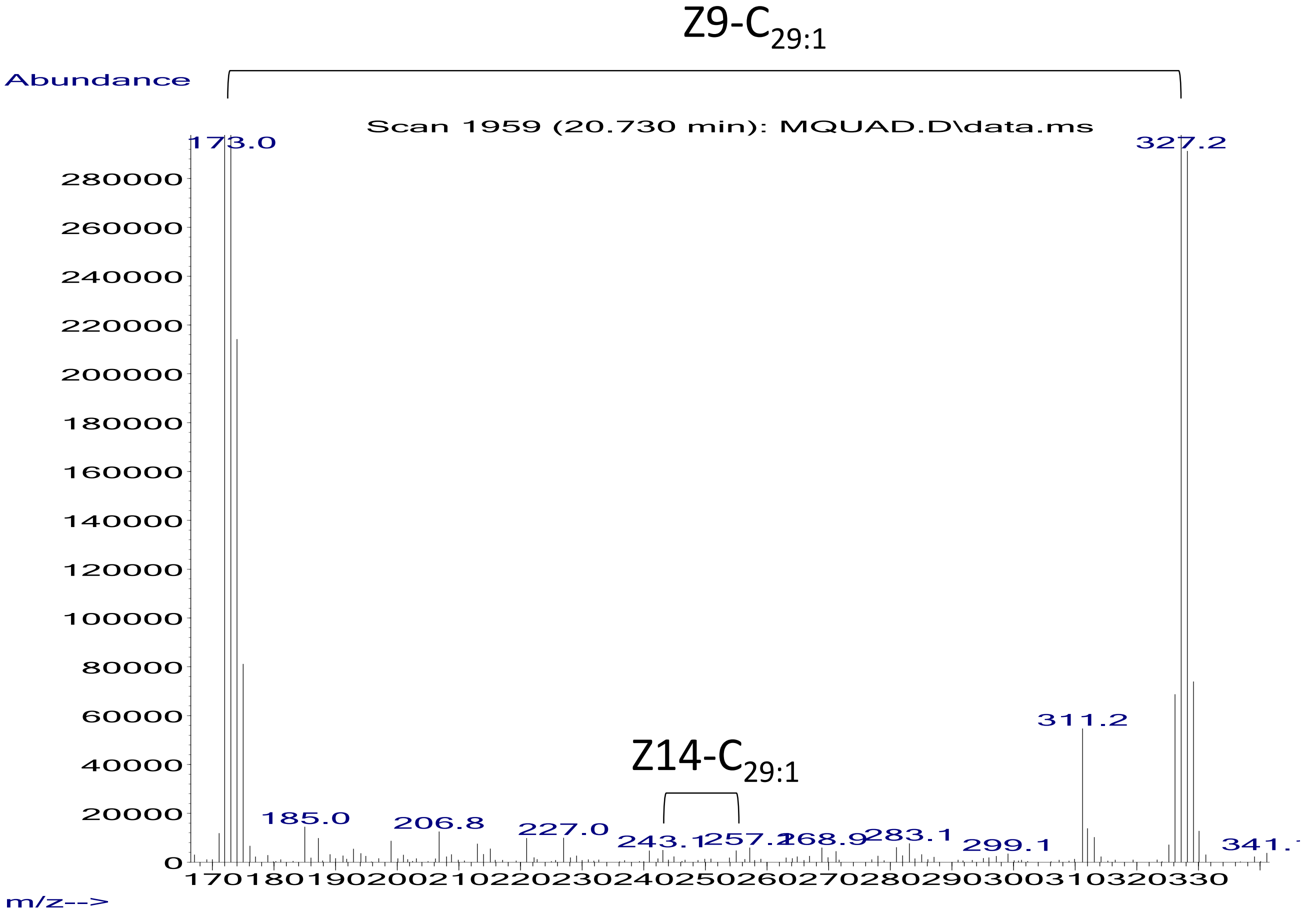

Fig. S4. The fragmentation patterns after a DMDS reaction showing the paired ions associated with the two alkene isomers detected in *M. quadrifasciata* collected from eastern Bahai in Brazil.
