# Supplementary material for: Are Isomeric Alkenes Used in Species Recognition among Neo-Tropical Stingless Bees (Melipona Spp)
Source: J Chem Ecol. 2017 Nov 17;43(11):1066–72. doi: 10.1007/s10886-017-0901-5 (PMC5735199; doi:10.1007/s10886-017-0901-5)
Supplement: Supplementary file 6 — (PDF 127 kb) [file 10886_2017_901_MOESM6_ESM.pdf]

# *M. asilvai*

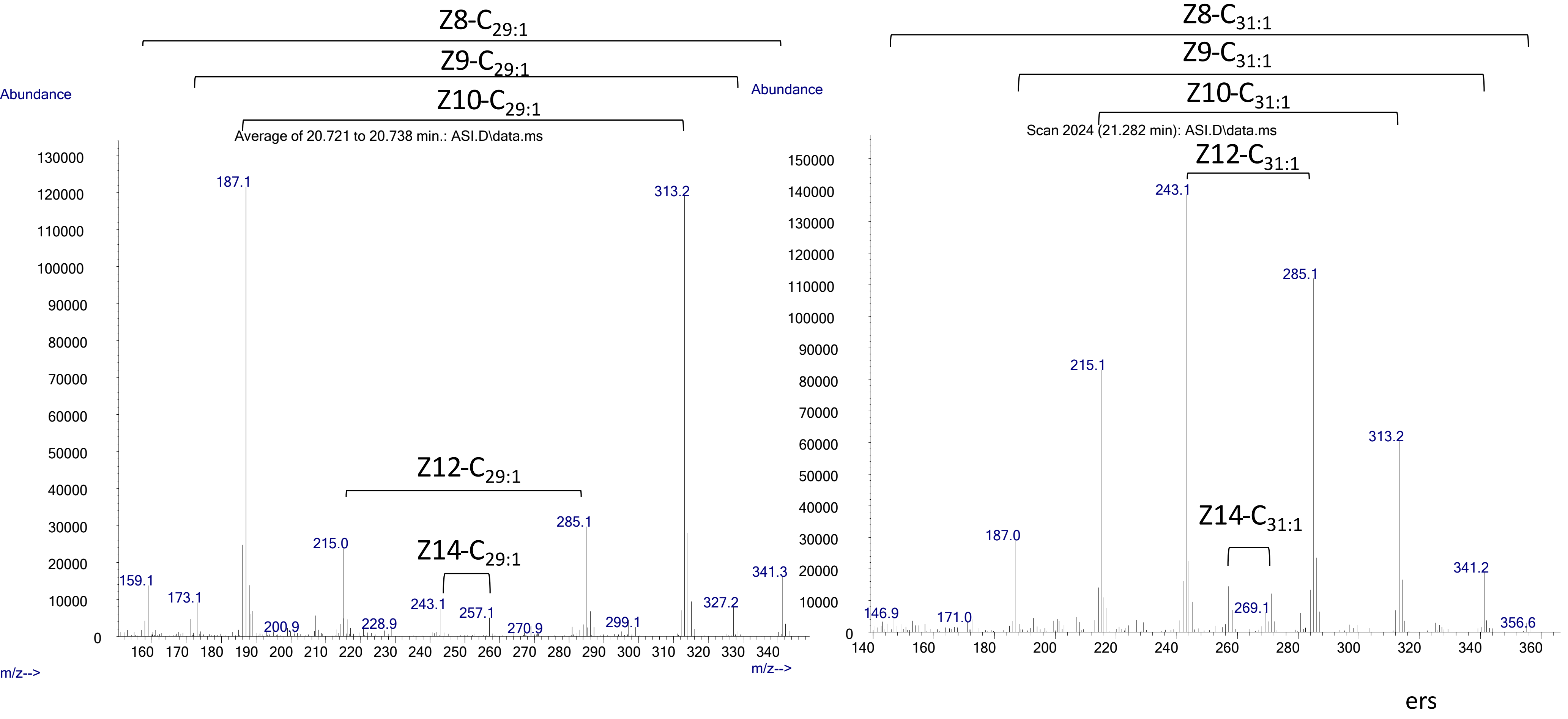


Fig. S6. The fragmentation patterns after a DMDS reaction showing the paired ions associated with the 5 different alkene isomers at two different chain lengths in *M. asilvai* collected from central Bahai in Brazil
