# Supplementary material for: Are Isomeric Alkenes Used in Species Recognition among Neo-Tropical Stingless Bees (Melipona Spp)
Source: J Chem Ecol. 2017 Nov 17;43(11):1066–72. doi: 10.1007/s10886-017-0901-5 (PMC5735199; doi:10.1007/s10886-017-0901-5)
Supplement: Supplementary file 7 — (PDF 73 kb) [file 10886_2017_901_MOESM7_ESM.pdf]

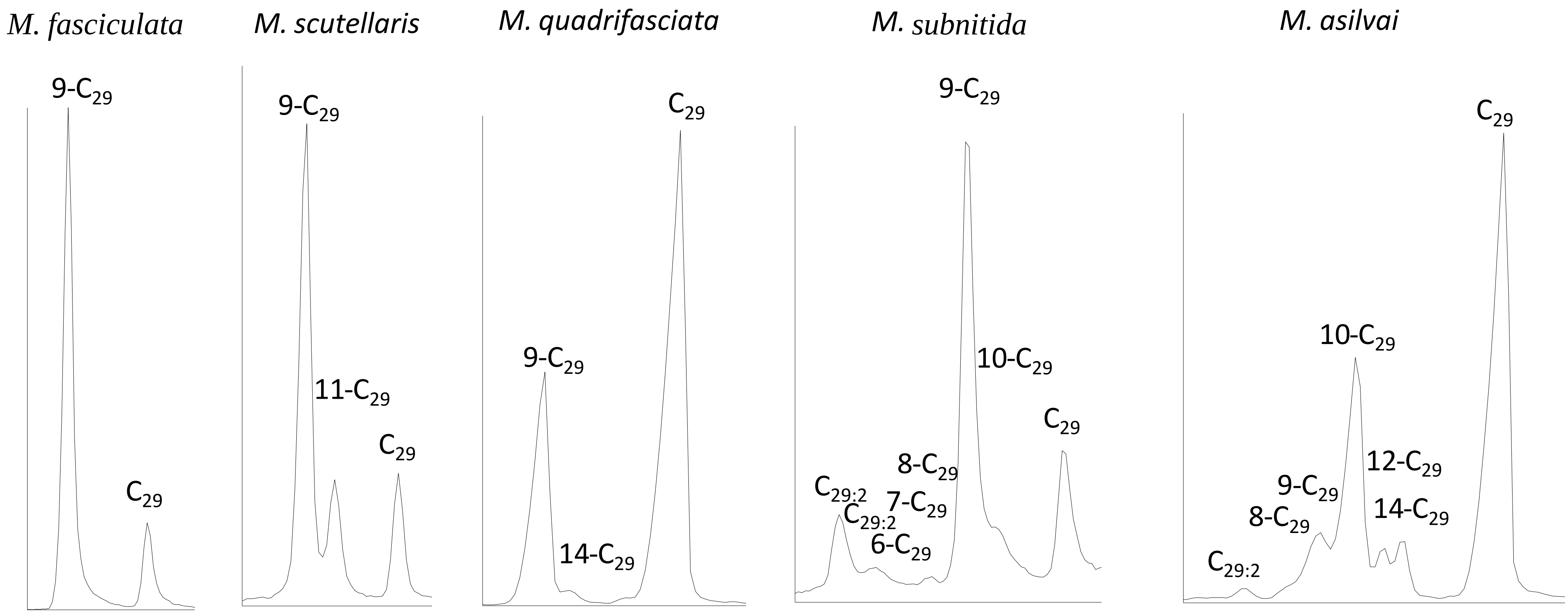

Fig. S7. A section of the TIC's for five species of *Melipona* and the corresponding C29 alkene isomers revealed by the DMDS reactions.
